# Mitochondrial DNA haplogroups and risk of transient ischaemic attack and ischaemic stroke: a genetic association study

**DOI:** 10.1016/S1474-4422(10)70083-1

**Published:** 2010-05

**Authors:** Patrick F Chinnery, Hannah R Elliott, Anila Syed, Peter M Rothwell, for the Oxford Vascular Study

**Affiliations:** a Mitochondrial Research Group, Institute for Ageing and Health and Institute of Human Genetics, Newcastle University, Newcastle upon Tyne, UK; b Stroke Prevention Research Unit, University Department of Clinical Neurology, John Radcliffe Hospital, Oxford, UK

## Abstract

**Background:**

Genetic factors have a role in the pathogenesis of ischaemic stroke, but the main genes involved have yet to be defined. Mitochondrial mechanisms have been implicated in the pathophysiology of acute stroke, but the role of mitochondrial DNA (mtDNA) has not been comprehensively studied. We investigated whether there is an association between mtDNA haplotypes and incidence of stroke.

**Methods:**

The major European mtDNA haplogroups were identified in two independent subpopulations (n=950) from a study of occurrence of transient ischaemic attack (TIA) and ischaemic stroke and were compared with those of patients with acute coronary syndromes from the same populations (n=340) and with those of independent population controls (n=2939).

**Findings:**

The presence of mtDNA sub-haplogroup K was significantly less frequent in patients with TIA or stroke than in controls in both subpopulations separately and in a pooled analysis (odds ratio 0·54, 95% CI 0·39–0·75, p<0·00001). This association remained highly significant after adjustment for multiple haplogroup comparisons. The association was significant for patients with TIA and stroke separately and was independent of known risk factors, but was not found for patients with acute coronary events. The mtDNA sub-haplogroup K was present in 8·7% of the total UK population controls and therefore confers a 4·0% (95% CI 2·2–5·7) reduction in population attributable risk of TIA and stroke.

**Interpretation:**

Genetic variation of mtDNA sub-haplogroup K is an independent determinant of risk of cerebral, but not coronary, ischaemic vascular events. These findings implicate mitochondrial mechanisms in the aetiology of ischaemic stroke and provide a new means for the identification of individuals with a high susceptibility of developing ischaemic stroke.

**Funding:**

Medical Research Council UK, National Institute of Health Research (NIHR), the Stroke Association, the Dunhill Medical Trust, the NIHR-funded Oxford Biomedical Research Centre, the NIHR-funded Newcastle Biomedical Research Centre in Ageing, and the Wellcome Trust.

## Introduction

Ischaemic stroke is a major cause of severe disability and premature death. Hypertension, hyperlipidaemia, diabetes, and smoking are all risk factors for stroke, but do not account for all of the risk.^1^ Findings from twin and family studies lend support to a role for inherited factors in both transient ischaemic attack (TIA) and ischaemic stroke,^2^ even after the exclusion of known monogenic causes, and a complex interplay between environmental and inherited factors is likely.^3^ Heritability of stroke seems to be greater in women than in men, with a two-fold excess of maternal versus paternal stroke in female probands but not in male probands.^4^ This difference could be caused by epigenetic or environmental factors, but is also consistent with a maternally transmitted risk allele. However, most genetic studies done so far in patients with stroke have focused on the nuclear genome, including several genome-wide association studies.^5^, ^6^, ^7^ Few studies have investigated the role of mitochondrial DNA (mtDNA), which is almost exclusively maternally inherited and could therefore account for the recent genetic epidemiological findings.

mtDNA is an appealing candidate for heritability of stroke for several other reasons. Pathogenetic mutations of mtDNA cause a maternally inherited mitochondrial disease characterised by stroke-like episodes (mitochondrial encephalomyopathy with lactic acidosis and stroke-like episodes) associated with biochemical and structural abnormalities of the cerebral vasculature.^8^ Patients carrying specific mtDNA point mutations are prone to stroke in the occipital regions. Additionally, genetic variation of mtDNA has been associated with several risk factors for ischaemic stroke, including hypertension,^9^ type 2 diabetes,^10^ increased LDL concentrations,^11^ obesity,^12^ and the metabolic syndrome.^13^

mtDNA has accumulated polymorphic variants, or single nucleotide polymorphisms (SNPs), throughout human history. In the absence of biparental recombination, these SNPs form branches of an evolving phylogenetic tree. The major subdivisions of the global mtDNA phylogeny occurred more than 10 000 years ago and are called mtDNA haplogroups. These haplogroups developed as human beings migrated into new geographic regions, leading to region-specific haplogroup variation. More than 95% of Europeans belong to one of ten major haplogroups, H, J, T, U, K (a subgroup of U), M, I, V, W, and X. Each haplogroup defines a clade of related mtDNAs that contains specific sequence variants within the population. mtDNA haplogroups affect the assembly and stability of the mitochondrial respiratory chain^14^ and the propensity to develop oxygen free radicals,^15^ which are implicated in the pathophysiology of ischaemic stroke.

Data from several small studies have indicated an association between different mtDNA haplogroups and different forms of ischaemic stroke;^16^, ^17^, ^18^ however, only one study included more than 500 individuals,^16^ none have replicated their findings in a separate cohort, and none fully accounted for potential confounding factors by intermediate phenotypes, particularly premorbid blood pressure. We investigated the mtDNA haplogroup distribution in two independent UK cohorts of patients with TIA or ischaemic stroke and compared them with healthy control individuals and patients with acute coronary syndromes, which share major risk factors with ischaemic stroke.

## Methods

### Participants

This case-control study included two independent disease cohorts and several independent control groups of white individuals within the UK.

The primary disease group included patients from the Oxford Vascular Study (OXVASC), a population-based study of patients with acute vascular events, including patients with TIAs, strokes, and acute coronary syndromes.^19^ This subpopulation was derived from two distinct populations: individuals resident in Oxford city and individuals resident in surrounding towns and villages in Oxfordshire.

The methods of OXVASC and details of the study populations have been reported previously.^19^ Briefly, all cases of TIA, ischaemic stroke or acute coronary syndrome were ascertained from an overall population of 91 000 individuals, irrespective of age, who were registered with 63 primary care physicians in four primary care practices in Oxford and in five practices in the surrounding towns and villages in Oxfordshire, UK. In the UK, almost all individuals register with a primary care practice, which holds a lifelong medical record. OXVASC was approved by a local research ethics committee and began on April 1, 2002, after a 3-month pilot study to ensure reliable case ascertainment. All patients with acute vascular events in the OXVASC population were identified by several overlapping methods, including prospective daily searches for acute events in hospital wards and clinics and retrospective searches of hospital and primary care administrative and diagnostic coding data. Direct assessment of ascertainment has shown it to be complete.^19^

Patients were eligible for inclusion in the present study if they had had a definite TIA or stroke (ie, cases) or an ST-elevation acute myocardial infarction (ie, disease controls) between April 1, 2002, and March 31, 2007. Patients were assessed by a study physician as soon as possible after the event in a hospital ward or in an outpatient clinic. Informed consent was sought, where possible, or assent was obtained from a relative. Clinical history, examination, and recording of investigations were standardised. Severity of stroke was assessed with the National Institutes of Health stroke scale (NIHSS). All cases were reviewed by the senior neurologist of the study (PMR) and classified as TIA, stroke, or other disorder by use of standard definitions.^19^ Patients were followed up by a research nurse or a clinical research fellow after 1, 6, 12, 24, and 60 months and were asked about any symptoms of recurrent ischaemic events. Recovery after stroke was assessed at these timepoints with the modified Rankin scale. All recurrent vascular events that presented to medical attention were also identified acutely by the continuing case ascertainment in the OXVASC study. Inpatients who moved out of the area were followed up by telephone.

If a recurrent vascular event was suspected at a follow-up visit or on reascertainment in OXVASC, the patient was reassessed and investigated by a study physician. The definitions of recurrent TIA and stroke used in OXVASC have been reported previously,^20^ and required a sudden new symptomatic neurological deterioration on a background of stability or improvement after the presenting event. Acute coronary events were defined by use of published criteria^21^ on the basis of history, electrocardiographic findings, cardiac biomarkers, autopsy, or death certificate. Troponin-I concentrations were measured with the Bayer Centaur assay, using the manufacturer's normal range (Bayer Healthcare Diagnostics Division, Tarrytown, NY, USA).

### Procedures and statistical analysis

The major European haplogroups were genotyped in five multiplexed allele-specific primer extension reactions by use of matrix-associated laser desorption/ionisation time of flight mass spectrometry (MALDITOF; MassARRAY, Sequenom, San Diego, CA, USA; webappendix) as described.^22^ 5% of the samples were randomly selected for confirmatory genotyping by use of a standard restriction enzyme approach,^23^ which yielded 100% concordance.

Haplogroup frequencies were compared by use of Fisher's exact test, and odds ratios with confidence intervals were calculated. The relation between the other variables and the haplogroups was studied by use of logistic regression analysis.

We initially compared the frequency of the major mtDNA haplogroups with the type of vascular event: TIA or ischaemic stroke (cases) versus ST-elevation acute myocardial infarction (disease controls). If patients with TIA or stroke had a previous myocardial infarction or had a myocardial infarction during follow-up they were included in the analysis as a TIA or a stroke case. Patients with myocardial infarction who had a history of stroke before the study period were not excluded.

We also compared the genotype frequencies with two ethnically matched UK control groups. The first group included 1500 healthy control individuals—1010 from the UK Medical Research Council 1958 birth cohort; 344 healthy neonates from north Cumbria, UK, and 146 healthy adult controls without previous TIA or stroke, recruited as part of the OXVASC study. The second group included 1439 disease control individuals—939 patients with type 2 diabetes from the Diabetes UK Warren 2 cohort,^24^ and 500 patients with amyotrophic lateral sclerosis.^25^ The haplogroup frequency of these two disease control groups did not differ from that of the general population.^24^

We first analysed all cases (ie, patients with TIA, stroke, or acute coronary syndromes) and controls from the Oxford subpopulation and then tested any associations in the Oxfordshire subpopulation. Any associations of specific haplotypes with TIA and ischaemic stroke that were significant in both subpopulations were then related to additional potentially relevant baseline and outcome data collected from the patients with TIA or ischaemic stroke: TIA versus stroke at baseline, age at first stroke, sex, diabetes, hypertension, atrial fibrillation, migraine, nature of the presenting event (stroke *vs* TIA), stroke severity (NIHSS score) at first assessment, affected vascular territory (anterior *vs* posterior circulation, brainstem *vs* occipital lobe), aetiological subtype of TIA or ischaemic stroke (classification from the Trial of Org 10172 in Acute Stroke Treatment [TOAST]), the main intermediate phenotypes (extent of symptomatic or asymptomatic carotid stenosis), leukoaraiosis on CT brain imaging, total cholesterol concentration at entry, stroke recovery (Rankin score at 6 months of follow-up), and the risk of recurrent stroke. A detailed record of family history of any acute vascular events in all first-degree relatives was obtained from the patient and/or relatives.

Given the strength of the epidemiological association between hypertension and risk of stroke,^26^ and the evidence of effects of mutations in mtDNA on blood pressure,^16^ we investigated any potential confounding of apparent genetic associations by blood pressure in particular detail. All premorbid measurements of blood pressure during the previous 10 years were extracted from primary care and hospital records for all patients with TIA or stroke in the OXVASC study and the following variables were calculated: mean, maximum, minimum, and standard deviation. Blood pressure was also measured at the time of the TIA or stroke and at 1, 6, and 12 months of follow-up. Analyses were done with and without stratification by antihypertensive medication.

### Role of the funding source

The funding sources had no role in the study design, in the collection, analysis, or interpretation of the data, or in the writing of the report. PMR had full access to all the data in the study. PMR and PFC had final responsibility for the decision to submit for publication.

## Results

1282 patients had TIA or stroke in the overall OXVASC population, of whom 1199 (723 stroke, 476 TIA) survived to clinical assessment and consented to providing a blood sample. 950 individuals had sufficient quality and quantity of DNA available for testing. Of 411 patients ascertained to have acute ST-elevation myocardial infarction, 386 consented to providing a blood sample, and DNA was available in 340 individuals. There were no significant differences in the type or severity of TIA or stroke, nor in the vascular risk factors, between the patients for whom a DNA sample was available and those for whom no DNA sample was available. Thus, the incomplete ascertainment of DNA samples was highly unlikely to have affected the findings of this study.

In the Oxford city subpopulation, the frequency of sub-haplogroup K was significantly lower in patients with TIA or stroke than in patients with acute coronary syndromes (p=0·0098), healthy UK controls (p=0·0082), and the combined control cohort of healthy and disease controls (p=0·0066). The frequency of all other major haplogroups did not differ significantly between patients with TIA or stroke and controls (table 1), including a comparison of the combined group of the rarer haplogroups I, W, V, X, M with other rare European and non-European haplogroups (p=0·96). In the Oxfordshire county subpopulation, we observed a similarly low frequency of sub-haplogroup K in patients with TIA or stroke compared with patients with acute coronary syndromes (p=0·049), healthy UK controls (p=0·0034), and the combined cohort of healthy and disease controls (p=0·0022). Combination of both OXVASC subpopulations increased the strength of the association; the frequency of sub-haplogroup K in patients with TIA or stroke was about half that in patients with acute coronary syndromes (p=0·0016), healthy UK controls (p=0·0002), and the combined cohort of healthy and disease controls (odds ratio 0·54, 95% CI 0·39–0·75, p<0·0001; table 2). The frequency of sub-haplogroup K in patients with TIA and in patients with stroke was significantly lower than in the UK population controls, but the frequencies of all other major haplogroups did not differ from the frequencies in controls (table 1).

**Table 1:** Frequency of the major European mitochondrial DNA haplogroups in the OXVASC study population and in controls

		**H**	**T**	**J**	**U**	K*	**I**	**W**	**V**	**X**	**M**	**Other**†	**Total**
**Cases**													
Oxford city subpopulation													
	TIA or stroke	215 (45·8%)	48 (10·2%)	42 (9·0%)	75 (16·0%)	24 (5·1%)	9 (1·9%)	14 (3·0%)	26 (5·5%)	6 (1·3%)	0 (0%)	10 (2·1%)	469
	ACS	56 (35·2%)	21 (13·2%)	17 (10·7%)	27 (17·0%)	18 (11·3%)	1 (0·6%)	2 (1·3%)	4 (2·5%)	1 (0·6%)	8 (5·0%)	4 (2·5%)	159
Oxfordshire subpopulation													
	TIA or stroke	225 (46·8%)	50 (10·4%)	53 (11·0%)	72 (15·0%)	23 (4·8%)	8 (1·7%)	10 (2·1%)	23 (4·8%)	9 (1·9%)	1 (0·2%)	7 (1·5%)	481
	ACS	82 (45·3%)	20 (11·1%)	16 (8·8%)	26 (14·4%)	16 (8·8%)	2 (1·1%)	3 (1·7%)	4 (2·2%)	1 (0·6%)	9 (5·0%)	2 (1·1%)	181
OXVASC total													
	TIA or stroke	440 (46·3%)	98 (10·3%)	95 (10·0%)	147 (15·5%)	47 (5·0%)	17 (1·8%)	24 (2·5%)	49 (5·2%)	15 (1·6%)	1 (0·1%)	17 (1·8%)	950
	ACS	138 (40·6%)	41 (12·1%)	33 (9·7%)	53 (15·6%)	34 (10·0%)	3 (0·9%)	5 (1·5%)	8 (2·4%)	2 (0·6%)	17 (5·0%)	6 (1·8%)	340
**Controls**													
Healthy UK controls		642 (42·8%)	157 (10·5%)	162 (10·8%)	187 (12·5%)	133 (8·9%)	43 (2·9%)	27 (1·8%)	54 (3·6%)	22 (1·5%)	16 (1·1%)	57 (3·8%)	1500
Disease controls		650 (45·2%)	129 (9·0%)	175 (12·2%)	202 (14·0%)	124 (8·6%)	34 (2·4%)	27 (1·9%)	37 (2·6%)	27 (1·9%)	1 (0·1%)	33 (2·3%)	1439
Total controls		1292 (44·0%)	286 (9·7%)	337 (11·5%)	389 (13·2%)	257 (8·7%)	77 (2·6%)	54 (1·8%)	91 (3·1%)	49 (1·7%)	17 (0·6%)	90 (3·1%)	2939

Data are n (%). OXVASC=Oxford Vascular Study. TIA=transient ischaemic attack. ACS=acute coronary syndrome.

* Haplogroup K is a subtype of U. The haplogroup U data do not include the K subgroup.

† Rare European and non-European haplogroups. Some numbers were rounded so not all percentages total 100%.

**Table 2:** European mitochondrial DNA haplogroups in the total OXVASC study population with acute transient ischaemic attack or stroke versus controls

	**Odds ratio**	**95% CI**
H	1·10	0·95–1·27
T	1·07	0·84–1·36
J	0·86	0·67–1·09
U	1·20	0·98–1·47
K*	0·54	0·39–0·75
I	0·68	0·40–1·15
W	1·38	0·85–2·25
V	1·70	1·19–2·43
X	0·95	0·53–1·70
M	0·18	0·02–1·36
Other†	0·58	0·33–1·00

Odds ratios were calculated from 950 cases and 2939 controls.

* Haplogroup K is a subtype of U. The data for the haplogroup U do not include the K subgroup.

† Non-European haplogroups.

In logistic regression analyses, we found no significant associations between the frequency of sub-haplogroup K and any of the following baseline characteristics: age at first TIA or stroke, sex, diabetes, hypertension, atrial fibrillation, migraine, and previous use of aspirin or statins. There was also no significant heterogeneity in the association in relation to stroke severity (NIHSS score at first assessment), the affected vascular territory (anterior *vs* posterior circulation; brainstem *vs* occipital lobe), the aetiogical subtype of ischaemic stroke (TOAST classification), or the main intermediate phenotypes (extent of symptomatic or asymptomatic carotid stenosis, leukoaraiosis on CT brain imaging, or total cholesterol concentration at entry). Frequency of sub-haplotype K was also unrelated to stroke recovery (Rankin score at 6 months' follow-up).

Among patients with TIA or stroke, sub-haplogroup K was not significantly associated with systolic or diastolic blood pressure at the time of first assessment: mean (SD) systolic blood pressure for patients with sub-haplogroup K was 152·3 mm Hg (26·3) versus 156·1 mm Hg (29·7) for patients with other sub-haplotypes (p=0·38), and mean (SD) diastolic blood pressure was 84·1 mm Hg (13·7) for those with sub-haplogroup K versus 84·5 mm Hg (14·7) for those with other sub-haplotypes (p=0·83). Both groups had an average of 18 premorbid blood pressure measurements, but there were no significant differences in mean systolic or mean diastolic blood pressure, or in minimum, maximum, or SD systolic or diastolic blood pressure. For example, mean (SD) premorbid systolic blood pressure in individuals with sub-haplogroup K was 144·3 mm Hg (13·1) versus 147·4 mm Hg (15·6) in individuals with the other sub-haplotypes (p=0·18). There were also no differences in systolic or diastolic blood pressure on follow-up. Stratification of these analyses by use of antihypertensive drugs did not reveal any significant differences between mtDNA haplotypes.

To assess the effect of sub-haplogroup K at the population level, we determined the population attributable risk, which depends on the relative risk of sub-haplogroup K and the proportion of the population who carry sub-haplogroup K.^27^ Based on the data we present here, the reduction in population attributable risk of TIA and ischaemic stroke conferred by sub-haplogroup K is 4·0% (95% CI 2·2–5·7).

## Discussion

Our findings show a strong association between mtDNA sub-haplogroup K and reduced risk of TIA and ischaemic stroke in the UK population. This association is not present in patients with acute coronary syndromes and was not explained by confounding by blood pressure or other intermediate phenotypes. This result is consistent with the hypothesis that genetic variation of mtDNA is a risk factor specifically for cerebral ischaemic events that are independent of the known major vascular risk factors.

How should we interpret our findings, given the growing amount of publications associating different mtDNA haplogroups with many complex traits? First, our initial association was confirmed in a second subpopulation, was present both in patients with TIA and in patients with ischaemic stroke, and was based on a comparison with several control groups, thus reducing the likelihood of a false-positive result. Second, disease controls with an acute coronary syndrome were collected in parallel, did not show the association of the haplotype with disease, and had a genotype distribution indistinguishable from 2939 UK controls. Third, we confirmed the findings of the largest previous studies, which did not identify an association between mtDNA haplogroups and ischaemic heart disease, hypertension, diabetes, or other features of the metabolic syndrome.^28^ Fourth, the difference in the frequency of sub-haplogroup K remained highly significant even after a conservative Bonferroni correction for the multiple significance testing for the 12 different groups of European mtDNAs. Fifth, logistic regression analysis showed that the association was independent of known major cerebrovascular risk factors, despite extracting particularly detailed data on premorbid blood pressure—the strongest risk factor for TIA and stroke. Finally, our findings are in agreement with those of a previous phylogenetic study that identified clusters of sub-haplogroup K in 14 patients with occipital stroke.^18^ Furthermore, our data also complement two single-cohort studies: one in Japan^17^ for the eastern Asian haplogroup A, which is not present in European populations, and another in Portugal for the sub-haplogroup H1.^16^ This study was designed to investigate all of haplogroup H, and did not select specific subgroups such as H1.

Although data from a Danish study of 9254 individuals showed no association between haplogroup K1 and stroke or cardiovascular disease,^29^ only 439 of the participants had an ischaemic stroke, based on case-notes review. The phenotyping in our 950 study patients with TIA or ischaemic stroke was based on the direct clinical assessment of each case by the study team, using established diagnostic criteria and with a 95% rate of brain imaging or autopsy.^19^ Greater statistical power and diagnostic accuracy in our study than in the Danish study could possibly explain the discrepancy.

Given the epidemiological evidence of an increased risk of stroke among maternal relatives,^4^ we were surprised not to find an association between the maternal history of stroke and sub-haplogroup K. However, a meta-analysis of sex differences in heritability of ischaemic stroke^4^ showed that the risk of stroke is greater in the sisters of women with stroke than in the brothers of women with stroke, despite the fact that all siblings share the same mtDNA inherited from their mother. Thus, both the genetic data from this study (data not presented here), and previously published epidemiological data,^4^ indicate that the increased heritability of stroke in women is not likely to be exclusively mediated through mtDNA. Other explanations for the gender bias include sex-specific autosomal genetic factors, epigenetic, or non-genetic mechanisms.^4^

Previous studies have shown a decreased frequency of sub-haplogroup K in patients with idiopathic Parkinson's disease.^30^, ^31^ This association with the sub-haplotype K seemed to be specific to Parkinson's disease,^32^ suggesting that the association was not mediated through a common neurodegenerative process. Similarly, we saw no association with severity of stroke or with propensity for post-stroke recovery. This finding suggests that the mtDNA haplogroup effect in stroke is mediated through the pathophysiology of the acute event itself, independent of blood cholesterol concentration, diabetes, blood pressure, age, or sex. How could this occur?

There is emerging evidence indicating the functional consequences of mtDNA haplogroup variation at the biochemical level. Neutral genetic variants in mice are associated with increased production of reactive oxygen species,^33^ and mtDNA haplogroups in human beings modulate the assembly kinetics of mitochondrial respiratory chain proteins.^14^ This idea provides an explanation for the increased risk of visual failure in individuals with Leber's hereditary optic neuropathy, which is also linked to increased production of reactive oxygen species.^34^ Both mitochondrial dysfunction and oxidative stress contribute to vascular endothelial senescence^35^ and atherosclerosis.^36^ If this is the mechanism underpinning the haplogroup association we describe here, we cannot explain why the effect is specific for cerebrovascular disease. Moreover, further work is required to define the precise genetic variants or cluster of variants that give rise to the reduced risk of TIA and ischaemic stroke conferred by sub-haplogroup K and the precise mechanism of action.

Recent genome-wide association studies have not yet identified new genes associated with ischaemic stroke. A few SNPs have only fairly weak associations, with odds ratios conferring less risk than mtDNA sub-haplogroup K. Our findings indicate the importance of mtDNA as a genetic risk factor for stroke, adding weight to the growing body of evidence implicating mitochondrial mechanisms in the pathophysiology of the disorder, and providing a means to identify individuals at risk of ischaemic stroke in the general population.

## Web Extra Material

Supplementary webappendix
