# Supplementary material for: Mitochondrial DNA haplogroups and risk of transient ischaemic attack and ischaemic stroke: a genetic association study
Source: Lancet Neurol. 2010 May;9(5):498–503. doi: 10.1016/S1474-4422(10)70083-1 (PMC2855429; doi:10.1016/S1474-4422(10)70083-1)
Supplement: Supplementary webappendix [file mmc1.pdf]

THE LANCET Neurology

Supplementary webappendix

This webappendix formed part of the original submission and has been peer reviewed. We post it as supplied by the authors.

Supplement to: Chinnery PF, Elliott HR, Syed A, Rothwell PM; for the Oxford Vascular Study. Mitochondrial DNA haplogroups and risk of transient ischaemic attack and ischaemic stroke: a genetic association study. *Lancet Neurol* 2010; published online April 1. DOI:10.1016/S1474-4422(10)70083-1.

Table 1: **Polymorphisms screened by Sequenom MALDI-TOF for haplogroup allocation. Detailed methodology has been described previously.[1]**

Haplogroup	Polymorphism	Plex	Identification Key	
H	m.7028C>T	A	Haplogroup H	7028=C
K sub-haplogroup	m.10550A>G	A	Haplogroup K	10550=G<br>12308=G
JT	m.4216T>C	A	Haplogroup J	4216=C<br>4917=A
T	m.4917A>G	A	Haplogroup T	4216=C<br>4917=G
W	m.8994G>A	A	Haplogroup W	8994=A
X	m.6221T>C	A	Haplogroup X	6221=C
UK	m.12308A>G	B	Haplogroup U	12308=G<br>10550=A
HV	m.14766T>C	C	Haplogroup V	14766=C<br>7028=T
I	m.4529A>T	D	Haplogroup I	4529=T
M	m.15043G>A	E	Haplogroup M	15043=A<br>8701=G
N	m.8701A>G	E	Haplogroup N	8701=A<br>15043=G

References

1 Elliott HR, Samuels DC, Eden JA, Relton CL, Chinnery PF. Pathogenic mtDNA mutations are common in the general population. *Am J Hum Genet* 2008; **80:** 254–60.
